# The zinc-finger bearing xenogeneic silencer MucR in α-proteobacteria balances adaptation and regulatory integrity

**DOI:** 10.1038/s41396-021-01118-2

**Published:** 2021-09-28

**Authors:** Jian Jiao, Biliang Zhang, Meng-Lin Li, Ziding Zhang, Chang-Fu Tian

**Affiliations:** 1 State Key Laboratory of Agrobiotechnology, and College of Biological Sciences, China Agricultural University, Beijing, China; 2 MOA Key Laboratory of Soil Microbiology, and Rhizobium Research Center, China Agricultural University, Beijing, China

**Keywords:** Bacterial genetics, Bacterial evolution, Symbiosis

## Abstract

Foreign AT-rich genes drive bacterial adaptation to new niches while challenging the existing regulation network. Here we report that MucR, a conserved regulator in α-proteobacteria, balances adaptation and regulatory integrity in *Sinorhizobium fredii*, a facultative microsymbiont of legumes. Chromatin immunoprecipitation sequencing coupled with transcriptomic data reveal that average transcription levels of both target and non-target genes, under free-living and symbiotic conditions, increase with their conservation levels. Targets involved in environmental adaptation and symbiosis belong to genus or species core and can be repressed or activated by MucR in a condition-dependent manner, implying regulatory integrations. However, most targets are enriched in strain-specific genes of lower expression levels and higher AT%. Within each conservation levels, targets have higher AT% and average transcription levels than non-target genes and can be further up-regulated in the *mucR* mutant. This is consistent with higher AT% of spacers between −35 and −10 elements of promoters for target genes, which enhances transcription. The MucR recruitment level linearly increases with AT% and the number of a flexible pattern (with periodic repeats of Ts) of target sequences. Collectively, MucR directly represses AT-rich foreign genes with predisposed high transcription potential while progressive erosions of its target sites facilitate regulatory integrations of foreign genes.

## Introduction

The distribution and abundance of organisms can be proximally explained in the scenario of the multi-dimensional niche while its ultimate explanation demands evolutionary understanding [1]. The match between organisms and niches is described by “adaptation” that involves past genetic changes in the scenario of Darwin’s theory of evolution by natural selection [1]. The modern synthesis of evolution summarized by Julian Huxley in 1942 can be briefly outlined by its core tenet that “adaptive evolution” is a process, in which natural selection acts on heritable variability originating from accidental genetic changes, leading to increased frequency of advantageous variants and adaptation [2, 3]. This adaptive evolution framework is still the root of the current standard evolutionary theory which recognizes four evolutionary processes (selection, mutation, recombination, and genetic drift) and only considers selection as a directional force increasing organismal adaptation [3]. In order to handle fluctuating abiotic and biotic stimuli, free-living bacteria and those pathogens or symbionts facultatively associated with eukaryotes are common to have an open pangenome which is characterized with a relatively small set of core genes and a large proportion of accessory genes [4]. Accessory genetic materials of bacteria are mainly provided by extensive horizontal transfer events within microbiota, which raises a challenging issue for maintaining many foreign “junk DNA” before the integration of few beneficial ones into existing transcriptional regulation network during adaptive evolution [5]. Indeed, it has been estimated that fine-tuned integration of a new gene into the regulation network of *Escherichia coli* can take millions of years [6]. Moreover, lineage-specific functions have been extensively recruited in bacterial adaptation [7]. Therefore, the maintenance and recruitment mechanism of foreign genes in the regulation scenario is a key to understand adaptation, speciation and the “home life” of living organisms, though it is largely unexplored.

New foreign DNA regions including both coding and non-coding sequences are usually AT-rich compared to the genome average [8]. Convergently evolved xenogeneic silencers preferring and maintaining AT-rich sequences [9] have been identified using ﻿chromatin immunoprecipitation (ChIP) and transcriptomic analysis for several model bacteria, such as H-NS in *E. coli* [10, 11] and *Salmonella enterica* [12, 13], MvaT in *Pseudomonas aerugniosa* [14], Lsr2 in *Mycobacterium tuberculosis* [15], and Rok in *Bacillus subtilis* [16], but it has not been explored how these xenogeneic silencers are involved in adaptive integration of foreign genetic materials into the regulation network (hereafter referred as “adaptive regulation”). With their temporally or spatially circumscribed effects, regulatory mutations in non-coding sequences rather than changes in coding region have been considered as a more common process during adaptive evolution [5, 17]. This is consistent with various examples in bacteria showing a contrasting turnover of target genes of the same transcription regulator in different species/strains, allowing their differential adaptation to distinct niches [18]. Consequently, we hypothesize that adaptive evolution in regulatory non-coding sequences targeted by xenogeneic silencers may underscore the recruitment of foreign genes during adaptation to various niches. This hypothesis of adaptive regulation involving both evolutionary and regulatory scenario can be considered as an updated version of the “ancestral” static transcriptional repression of foreign junk DNA by xenogeneic silencers.

To investigate the bacterial recruitment mechanisms for foreign genes within this adaptive regulation framework, we focused on rhizobia which live saprophytically in soil and form nitrogen-fixing bacteroids in legume root nodules in the nitrogen-depleted soil [19, 20]. Recent coexpression network analysis of broad-host-range *Sinorhizobium fredii* under free-living and symbiotic conditions demonstrated that the more conserved a gene is (from strain-specific to genus core genes), the greater its average transcriptional level and connection degree [21]. This pattern implies possible adaptive, not neutral, evolution of pangenome. This species and other *Sinorhizobium* members are also characterized by their multipartite genomes composed of chromosome, chromid, symbiosis plasmid and variable number of smaller plasmids [21, 22], among which chromid ﻿has plasmid-type maintenance and replication systems while having sequence signatures more similar to chromosomes than plasmids [23]. The MucR/Ros family protein has been intensively studied in rhizobia, particularly *Sinorhizobium* [24–32], showing pleiotropic roles under both free-living and symbiotic conditions. MucR/Ros homologs are conserved in α-proteobacteria and have a zinc-finger motif [30, 33]. Recent studies demonstrate that MucR/Ros homologs show oligomerization and heat-stable features [34, 35], which are shared by the well-known xenogeneic silencer H-NS of *E. coli* [36]. Scattered evidence suggests that MucR homologs bind to AT rich regions in and around test bacterial promoters [9, 24, 26, 29, 37].

In this work, to investigate whether MucR is a global xenogeneic silencer and its potential role in adaptive regulation of foreign genes, ChIP-seq coupled with transcriptomic data were used to study *S. fredii* CCBAU45436 (SF45436) that is a microsymbiont of diverse legumes including cultivated and wild soybeans [21, 38, 39]. Adaptive regulation of MucR target genes belonging to individual pangenome subsets of different conservation levels (from genus core to strain-specific genes) or replicons (chromosome, chromid, symbiosis plasmid and two smaller accessory plasmids) were investigated in the wild-type and the *mucR* mutant under free-living and symbiotic conditions. The critical role of MucR in adaptive integration of foreign genes was discussed and highlighted in a working model in the context that AT-rich signature of foreign DNA can be progressively erased during adaptive evolution.

## Materials and methods

### Bacterial strains, plasmids, primers and growth conditions

*S. fredii* and *E. coli* strains, plasmids and primers used in this study are listed in Supplementary Table S1. *S. fredii* strains were grown at 28 °C in TY or YEM medium [40]. The growth of *S. fredii* strains in YEM broth were monitored by Bioscreen C (Oy Growth Curves Ab, Finland). *E. coli* was cultured in Luria-Bertani medium at 37 °C. Concentrations of antibiotics were 30 μg/mL for nalidixic acid, 50 μg/mL for kanamycin, and 30 μg/mL for gentamicin.

### Comparative genomics and COG function annotation

To define the pangenome subsets of SF45436, protein sequences from 12 *Sinorhizobium* genomes were clustered using the CD-HIT algorithm with a 70% sequence identity cut-off [41]. Hierarchical core/accessory genes were determined using the method described previously [21]. Briefly, four hierarchical subsets were defined as follows: Subset I (genus core of 12 *Sinorhizobium* strains), subset II (species core of 5 strains belonging to *S. fredii* excluding subset I), subset III (genes shared by three closely related *S. fredii* strains SF45436, SF25509 and HH103 but excluding subset I and II), and subset IV (SF45436-specific genes) [21]. COG annotation for SF45436 proteins was obtained using Reserved Position-Specific BLAST against COG database integrated in WebMGA [42].

### Chromatin immunoprecipitation sequencing (ChIP-seq)

The plasmid pBGST-MucR1 with a P*mucR1* promoting GST-MucR1 fusion protein was constructed based on pBBR1MCS-2 [43] and was conjugated into the *mucR1* mutant, resulting the gstMucR1_pla strain used in subsequent experiments. For bacteroid isolation, 5 g of soybean nodules (30 dpi) were crushed within an ice-cold mortar containing 20 ml of PBS added with 1% PVP (polyvinylpyrrolidone) and 1 g quartz sand. The homogenate was centrifuged at 4 °C, 400 × g for 10 min to remove quartz sand and plant tissue, and the supernatant was further centrifuged at 4 °C, 10,000 × g for 5 min to pellet bacteroid cells. Bacteroids or TY cultures (OD_600_ = 1.2) were resuspended in PBS (pH7.4), and formaldehyde was added to a final concentration of 1%. Crosslinking reactions were sustained for 15 min at room temperature with gentle shaking, and stopped by adding glycine to a final concentration of 0.125 M for 5 min. Crosslinked cells were ground into fine powder, resuspended with ChIP buffer (50 mM Tris-HCl (pH8.1), 150 mM NaCl, 5 mM EDTA, 1% Triton X-100, 0.1% sodium deoxycholate) plus protease inhibitor cocktail (CWbiotech). Cell lysates were sonicated on ice to shear DNA fragments to an average length of 300~400 bp and cleared by centrifugation at 14,000 × g for 15 min at 4 °C. Supernatants were normalized by protein content and pre-cleared with 40 μl proteinA/G agarose (CWbiotech). 10% of the supernatant was removed and used for total chromatin input DNA preperation. For each ChIP reaction, 5 μl monoclonal anti-GST (CWbiotech) was added and incubated overnight at 4 °C with 40 μl of protein-A/G agarose beads pre-saturated with BSA. The beads were washed twice with ChIP buffer, once with high salt buffer (50 mM Tris-HCl (pH8.1), 500 mM NaCl, 5 mM EDTA, 1% Triton X-100, 0.1% sodium deoxycholate) and LiCl buffer (10 mM Tris-HCl (pH 8.1), 25 mM LiCl, 0.5% sodium deoxycholate, 1 mM EDTA), and twice with TE buffer (10 mM Tris-HCl (pH 8.1) and 1 mM EDTA). Protein-DNA complexes were eluted in 500 μl elution buffer (1% SDS, 0.1 M NaHCO_3_), supplemented with NaCl to a final concentration of 300 mM and incubated overnight at 65 °C. Samples were treated with 0.5 mg of Proteinase K for 2 h at 45 °C. DNA was extracted using phenol:chloroform:isoamyl:alcohol (25:24:1), ethanol precipitated using Dr. GenTLE Precipitation Carrier (TAKARA). Library construction (300–400 bp) and deep sequencing (paired-end 125 using HiSeq 2500; Illumina) were performed by SinoGenoMax-Beijing, with the total chromatin input DNA as control. Three biological replicates were tested.

To investigate the potential side effects of plasmid borne GST-MucR1 in the gstMucR1_pla strain, a GST-MucR1 in situ expression strain, gstMucR1_chr, was constructed through homologous recombination by using pJQGST-MucR1 derived from pJQ200SK [44] carrying up- and down-stream homologous fragments of *mucR1* (Supplementary Table S1). Western blotting was carried out to analyze the specificity of anti-GST monoclonal antibody and to determine the accumulation of GST-MucR1 protein in gstMucR1_pla and gstMucR1_chr. These two strains were further compared with the wild type for their growth in YEM broth and plate, and symbiotic performance on host plants as previously described [21]. To verify the ChIP-seq data, seven representative peaks with different enrichment fold change values were tested by ChIP-qPCR with diluted DNA recovered from input and ChIP samples as templates. qPCR was performed by using QuantStudioTM 6 Flex and 2× RealStar Green Mixture (Genstar). ZnuA coding sequence [45] was used as the reference for normalization of MucR1 recruitment levels. Three independent biological replicates were analyzed.

### High-throughput data analysis

Clean ChIP-seq reads were mapped to the genome using bowtie2 to generate BAM files which were used for peak calling by running MACS2 (version 2.1.0) [46]. The parameters for peak and summit (peak center) calling are as follows: “macs2 callpeak --call-summits -B -g 6913799 --nomodel --extsize 100 --bw 300 --qvalue 0.01 --keep-dup 1 -f BAMPE --outdir MACS2_es100_bw300_BAMPE -t ChIP.bam -c Input.bam -n ChIP-Input”, in which “bw” and “extsize” represent bandwidth (half of the estimated sonication size, bp) and extension size (the minimum length of peaks, bp), respectively. Positive peaks (FDR < 0.01) with their summit distance less than 200 bp for different samples were re-allocated with the same peakID. A gene associated with a peak summit in its regulation region (−500 bp to +100 bp around the start codon) was assigned as target genes directly regulated by MucR1. Circos visualization of high-throughput data was accomplished by using ShinyCircos package in R [47]. When sequence features of peaks were analyzed, the central region of 200 bp (with summit in the peak center) was used.

Over-represented motif of ChIP-seq peaks was determined by analyzing the 200 bp central region using the web-based MEME-ChIP module. One of “class A flexible patterns” TTxxxGxxxTxxxxxxxxxxTT [48] highly coincides with the DNA-binding site resolved in a Ros87-DNA docking model [49] and was scanned for its distribution in individual replicons and genomic islands of SF45436. Motifs with no more than one insertion or deletion in the first two non-conserved regions and no more than two insertions or deletions in the third non-conserved region were recorded. Genomic islands were identified by using IslandViewer 4 with three different prediction methods: IslandPick, IslandPath-DIMOB, SIGI-HMM [50]. To summarize the distribution of this flexible pattern in SF45436 genome, the distance between the middle position of the motif and the first nucleotide of gene was used. This allowed us to get a general picture on the relative abundance of the flexible pattern along the promoter (boundary: −500 bp) and coding regions of genes. Since not all genes are the same length, the boundary for counting motifs within coding regions was set as +2000 bp. For those genes shorter than 2000 bp, their downstream intergenic regions were not included in the analysis to avoid interfering with true intragenic signals. Similarly, the distance between the summit position of ChIP-seq peak and the first nucleotide of gene was calculated to determine the relative abundance of ChIP-seq summits in the region from −500 bp to +100 bp. Kernel density (probability density) plots were then produced for visualizing these abundance data of the flexible pattern and ChIP-seq summits by using R package ggplot2 [51]. The number of class A flexible patterns and AT content were also calculated for the 200 bp central region of ChIP-seq peaks.

Oligonucleotide usage deviation values were calculated as described earlier [52]. Briefly, the zero-order Markov method is designed to determine the expected number of oligonucleotides by removing biases in mononucleotide frequencies. The normalized oligonucleotide deviation value for a word W is calculated by dividing the observed counts by the expected counts. MucR1 binding preference on each oligonucleotide was evaluated by Spearman’s rho between oligonucleotide usage deviations in the 200 bp central regions of ChIP-seq peaks and MucR1 recruitment levels.

Promoter sequences of *Sinorhizobium* identified previously [53] were used as input for the prediction of promoters identified by sigma factors RpoD, RpoH1, RpoH2, RpoE2, and RpoN in SF45436. The −35 and −10 elements of input promoters were extracted to construct separate ﻿position-specific scoring matrix. Profiles searching were performed on the SF45436 genome for putative promoter elements using PoSSuMsearch [54]. After profile searching, those putative promoters with the spacer length between −35 and −10 elements does not comply with the spacer length threshold were removed [53]. AT content of spacer sequences within the predicted intergenic and intragenic promoters was then calculated.

### Protein purification

The plasmids overexpressing His6-SUMO-tagged MucR1/MucR2 were constructed by cloning *mucR1/mucR2* into pET30a-SUMO. Soluble His_6_-SUMO, His_6_-SUMO-MucR1 and His_6_-SUMO-MucR2 were purified from *E. coli* BL21 (DE3) containing pHis-MucR1 and pHis-MucR2, respectively, under native conditions. Briefly, 500 ml culture was treated with 0.2 mM IPTG at 16 °C overnight. Then the cells were collected, resuspended in lysis buffer (25 mM Tris-HCl, pH 8.0, 250 mM NaCl, 50 mM imidazole, 5% glycerol) and lysed by ultrasonic shear until the suspension turned to be clear. After centrifugation at 250,000 × g the supernatant was loaded onto a chromatography column filled with 1 ml Ni-nitrilotriacetic acid resin (Ni-NTA, Qiagen). After washing with the same buffer of 5–10 fold volumes, the recombinant proteins were eluted using the elution buffer (lysis buffer supplemented with 500 mM imidazole) with a linear gradient. The elution fractions were purified by size-exclusion chromatography using a Superdex 200 10/30 column (GE Healthcare) and the SEC buffer (20 mM Tris–HCl, pH 8.0, 250 mM NaCl, 5% glycerol) and were divided into aliquots and stored at −80 °C.

### Electrophoretic mobility shift assays (EMSA)

The 250 bp promoter region of *visN* covering MucR1-binding sites was amplified in a PCR. The 5’-Cy5-labeled PCR fragments were purified and used as probes. The EMSAs were performed as follows: for each reaction, 12.3 nM Cy5-labeled DNA probe and various concentrations of test proteins were diluted into the binding buffer (25 mM Tris (pH7.5), 100 mM KCl, 5 mM MgCl_2_, 5% glycerol and 0.05% dodecyl maltoside, 0.5 mg/mL BSA, 0.1 mg/mL sonicated salmon sperm DNA) in a final volume of 10 μl. The samples were incubated at room temperature for 20-30 min, then separated on a 6% TB polyacrylamide gel (no EDTA) and the gel was scanned with a Typhoon FLA 9000 imager (GE Healthcare).

## Results and discussion

### ChIP-seq uncovers MucR1 as a global DNA-binding protein

**Fig. 1:**
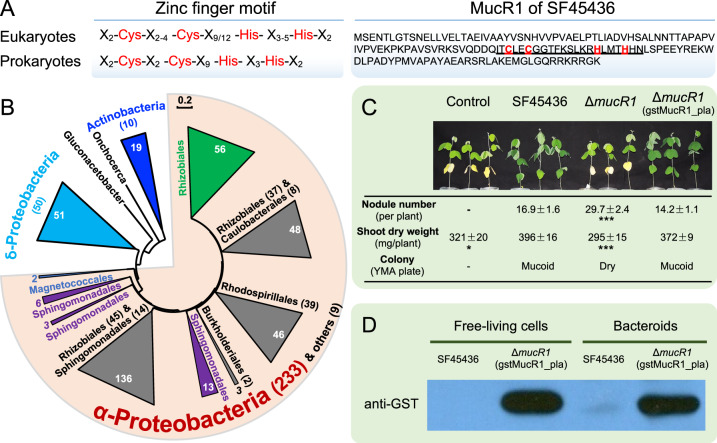
The zinc-finger regulator MucR1 in soybean microsymbiont SF45436. **A** MucR1 contains a conserved C2H2 zinc-finger motif. **B** The neighbor-joining phylogenetic tree of MucR/Ros homologs of the *ROS_MUCR* (PF05443) family proteins showing their enrichment in α-Proteobacteria. 273 Pfam seed sequences and 41 sequences from representative lineages not well covered by seed sequences were analyzed. The number of sequences from strains of corresponding phylum/class/order is shown in brackets. **C** The symbiotic and free-living defects of Δ*mucR1* can be recovered by introducing pBGST-MucR1. The P*mucR1* was used to drive the expression of GST-MucR1. Average values and standard error of the mean are shown. Significant difference between the mean of each treatment and that of SF45436 is indicated (*t* test; *, *P* value < 0.05; ***, *P* value < 0.001; more than eight plants were scored). **D** Detection of GST-MucR1 in free-living cells and bacteroids from soybean nodules by Western blot using anti-GST antibody.

We have demonstrated that MucR1 of SF45436, rather than its paralog MucR2 carrying a frameshift mutation, is essential in forming mucoid colonies and nitrogen-fixing nodules on soybean plants [31]. MucR1 in SF45436 has the conserved prokaryotic zinc-finger motif X_2_-Cys-X_2_-Cys-X_9_-His-X_3_-His-X_2_ (Fig. 1A) that is a characterized feature of *ROS_MUCR* (PF05443) family protein widely distributed in α-proteobacteria (Fig. 1B). To determine the direct targets of MucR1 in SF45436 genome, a GST-MucR1 expressing strain driven by the promoter of *mucR1* was constructed in the background of the *ΔmucR1* mutant. Similar to the wild-type SF45436, the resulting strain formed mucoid colonies and nitrogen-fixing nodules on soybean plants (Fig. 1C), indicating that the N-terminal fused GST-tag had no significant influence on the function of MucR1. Western blot using both free-living cells and symbiotic bacteroids demonstrated the specificity of monoclonal anti-GST antibody (Fig. 1D) that was used in subsequent ChIP-seq experiment.

**Fig. 2:**
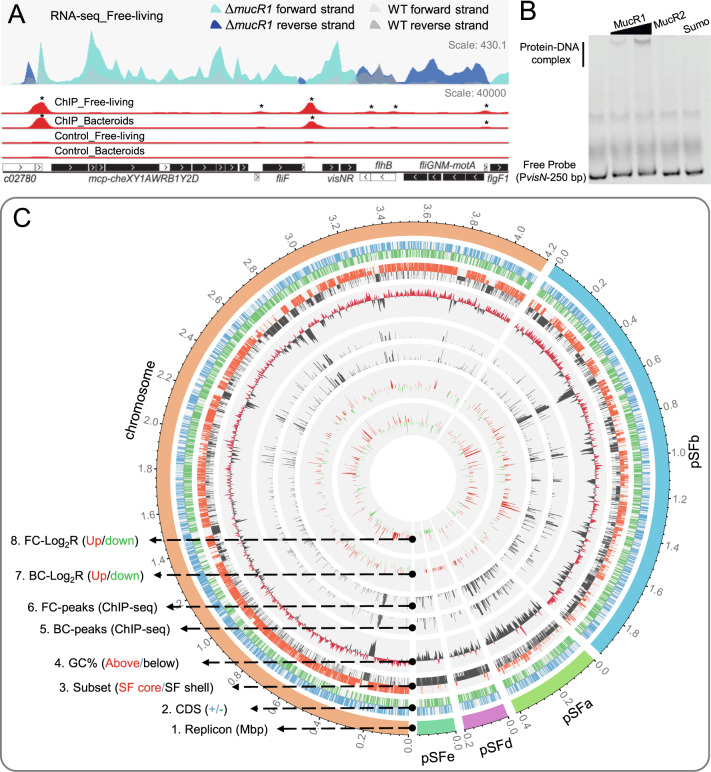
Mapping ChIP-seq and RNA-seq reads on the multipartite genome. **A** Representative figure showing coverage of reads from ChIP-seq (both free-living cells collected at OD_600_ = 1.2 in TY medium and bacteroids) on genetic loci involved in flagellum-dependent motility and chemotaxis. MucR1 ChIP-seq peaks identified are indicated by asterisks (Enrichment fold > 1, FDR < 0.001). Coverage of reads from strand-specific RNA-seq (free-living cells collected at OD_600_ = 1.2 in TY medium) is also shown. DEGs between SF45436 and the *mucR1* mutant are indicated as black box (log_2_Ratio(*mucR1/*SF45436) > 1.0, FDR < 0.001). **B** Electrophoretic mobility shift assay showing the direct binding of His_6_-SUMO-MucR1 (13.5 and 27 µM) on the promoter region of *visN* in vitro. His_6_-SUMO-MucR2 (27 µM) and His6-SUMO (27 µM) were negative controls. **C** Circos overview of the genome information and ChIP-seq/RNA-seq mapping results. 1. Replicons in different colors. 2. CDS on the “+” strand and “−” strand in blue and green color, respectively. 3. Genes within four hierarchically divided core/accessory subsets represented by different bars: Subset I (genus core; red/long), subset II (*S. fredii* core excluding subset I; red/short), subset III (genes shared by SF45436, SF25509 and HH103 but excluding subset I and II; black/short), subset IV (SF45436-specific genes; black/long). 4. GC% with window size of 1 kb with red and black color showing the value above and below the whole genome average, respectively. 5–8. BCs-peaks (bacteroids), FCs-peaks (free-living cells), log_2_Ratio(Δ*mucR1*/SF45436) in bacteroids and the free-living state are sequentially shown from the 5th to the 8th rings. Up- and down-regulated genes are indicated by red and green color on the 7th and 8th rings.

Previous RNA-seq analysis revealed MucR1 as a pleiotropic regulator under both free-living (TY medium, OD_600_ = 1.2) and symbiotic conditions (nodules from cultivated soybean JD17, 30 days post inoculation [DPI]) [31]. In this work, ChIP-seq was performed for both free-living cells (FC; TY medium, OD_600_ = 1.2) and bacteroid cells (BC; nodules from cultivated soybean JD17, 30 DPI) under the same conditions. Representative ChIP-seq peaks observed in a gene cluster involved in flagellum-dependent motility and chemotaxis are shown in Fig. 2A. Electrophoretic mobility shift assay verified that MucR1 can directly bind its target sequences such as the promoter region of *visN* (Fig. 2B) while MucR2 carrying a frameshift mutation cannot. 84% (1154) of FC peaks and 85.9% (806) of BC peaks were reproducibly uncovered in all three biological replicates, and 91.6% (1258) of FC peaks and 94.5% (886) of BC peaks were observed in at least two biological replicates (Supplementary Fig. S1A; *q*-value < 0.01). When the data from three biological replicates were combined, 1484 and 1068 significant peaks were identified (*q*-value < 0.01) in FC and BC samples, respectively (Fig. 2C and Supplementary Table S2). These peaks constitute a set of 1551 unique ChIP-seq peaks, of which 1002 were detected under both free-living and symbiotic conditions (Supplementary Table S2). Notably, there is a strong positive correlation between the enrichment fold values of shared ChIP-seq peaks under two conditions (Supplementary Fig. S1B; Pearson’s rho = 0.90, *P* value = 2.2e−16) whereas the enrichment fold values of condition-specific ChIP-seq peaks are generally low (531/549 below 5), such as those shown in Fig. 2A. This phenomenon is well presented in a Circos overview of ChIP-seq mapping patterns which are indistinguishable between FC-peaks and BC-peaks (Fig. 2C). These results indicate that the recruitment levels of MucR1 to most of its targets are not altered by two contrasting conditions, though targets specific to either free-living cells or bacteroids can also be detected implying potential condition-dependent regulation. This is in line with the pleiotropic role of MucR homologs in environmental adaptation of α-proteobacteria [31, 37, 55] and condition-modulated interactions of xenogeneic silencers with DNA [56].

By associating the peaks with nearby genes, totally 1350 protein encoding genes were defined as direct MucR1 target genes. Within these target genes, 1307 and 911 genes were found in FC and BC ChIP-seq experiments, respectively, and 868 genes are shared by two conditions (Supplementary Table S2). Noteworthy, these numbers of direct target genes are very conservative, considering that genes downstream of the first gene within a polycistronic operon were not included (such as those polycistrons in Fig. 2A).

To test if there was potential influence on the identification of ChIP-seq peaks caused by the expression level of plasmid borne GST fusion MucR (gstMucR1_pla), a derivative carrying a chromosomal borne GST fusion MucR (gstMucR1_chr) was constructed. Despite a slightly higher expression level of GST fusion MucR in the gstMucR1_pla strain compared to the gstMucR1_chr strain (Supplementary Fig. S2), seven representative ChIP-seq peaks of various enrichment levels identified in the gstMucR1_pla strain were also identified in the gstMucR1_chr strain in ChIP-qPCR (Supplementary Fig. S2). Moreover, the free-living and symbiotic phenotypes (Supplementary Fig. S2 and Supplementary Table S3) are similar between two strains. Therefore, the ChIP-seq results obtained in the gstMucR1_pla are reproducible.

### MucR1 directly regulates AT-rich core genes involved in stress and symbiosis adaptation

**Fig. 3:**
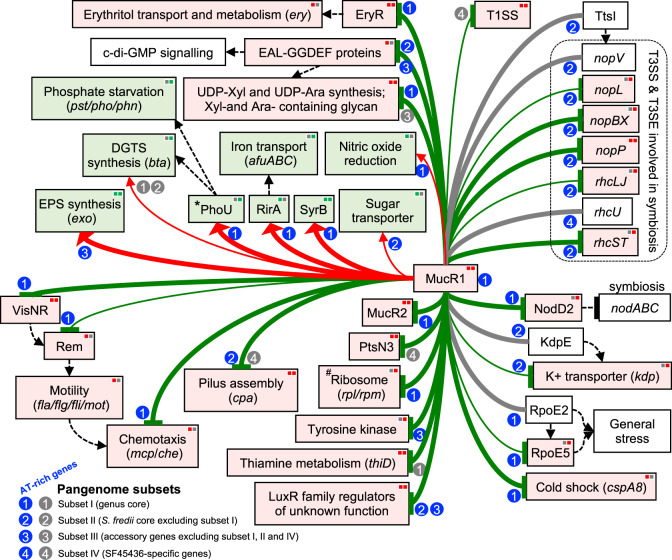
MucR1 directly regulates AT-rich functional genes of different conservation levels. Summary of MucR1 target genes and related expression profiles. Red and green solid lines indicate activation and repression, respectively, by MucR1. Thick and thin solid lines represent peaks enriched by the fold above 5 and the fold between 1 and 5, respectively. MucR1-dependent expression is indicated by the background color of boxes: pink (up-regulated in the *mucR1* mutant) and light green (down-regulated in the *mucR1* mutant). Arrows and “T” shape lines in black color show the activation and repression effects documented in literatures. DEGs detected in different conditions are indicated by different colors (red, Δ*mucR1*/WT, up-regulated; green, Δ*mucR1*/WT_down-regulated; left, free-living cells in TY medium at OD600 = 1.2; right, JD17 bacteroids). *, a strong recruitment signal was detected within ORF of *pstB* which is located in the *pstSCAB-phoUB* operon. #, differentially expressed genes were *c11230* and *c11240* encoding ribosomal proteins L16p and L29p. Numbers in circles indicate four pangenome subsets of different conservation levels, and blue circles indicate that these genes are AT-rich in corresponding pangenome subsets.

MucR1 negatively regulates several key functions involved in stress and symbiosis adaptation (Fig. 3). For example, *rpoE5* encodes a sigma factor responding to general stress; [57, 58] *visNR*, *rem* and several gene clusters encode transcriptional factors or functional components involved in motility (*fla/flg/fli/mot*), chemotaxis (*mcp/che*) and pilus assembly (*cpa*); [59, 60] *nodD2* encodes a negative regulator of nodulation genes in *S. fredii*; [61, 62] a gene cluster encodes structure components of T3SS (type three secretion system) and the effector NopP modulating compatibility of *S. fredii* with legume hosts [63–65]. On the other hand, there are few processes subject to positive regulation by MucR1 (Fig. 3), such as *exo* genes involved in the biosynthesis of ﻿succinoglycan exopolysaccharide [24], *phoUB* regulating phosphate starvation machinery [66, 67], and *rirA* coding an iron responsive regulator required for effective symbiotic nitrogen fixation of *S. fredii* on soybean plants [68]. Among few studies of MucR-DNA interactions, evidence for direct transcriptional activation role of MucR is so far just available for *exoY* encoding a galactosyltransferase that initiates the assembly of repeating unit of ﻿succinoglycan exopolysaccharide [24]. These well-characterized functional target genes have higher AT than the average of individual pangenome subsets and most of them belong to genus or species core genes (Fig. 3; blue circles with numbers 1 and 2). These findings suggest MucR1 as a master regulator for these AT-rich core genes providing adaptation benefits under either free-living or symbiotic conditions, indicating successful regulatory integrations. For example, a MucR-repressed cryptic gene cluster directing exopolysaccharide production can be activated by a local regulator CuxR in the presence of c-di-GMP in *Sinorhizobium meliloti* [32, 69].

### MucR1 target genes are enriched in less conserved strain-specific foreign genes

**Fig. 4:**
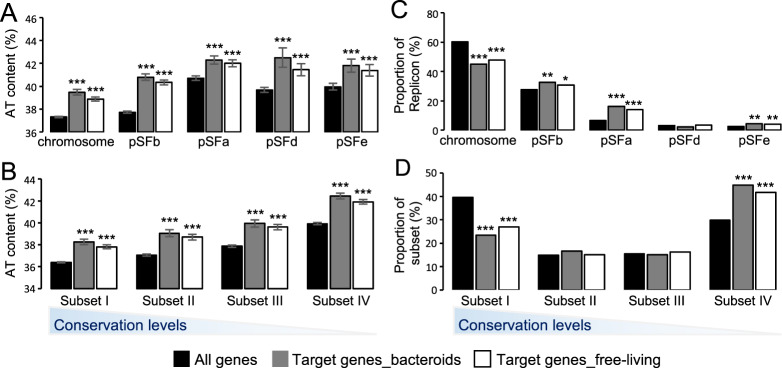
MucR1 targets across subsets of different conservation levels and replicons. AT% content of peak-associated genes within individual replicons (**A**) and different core/accessory subsets (**B**). Error bars represent standard error of the mean. Significant difference between target AT% and the corresponding average value (black column) of the same subset or replicon is indicated (*, *P* value < 0.05; **, *P* value < 0.01; ***, *P* value < 0.001; *t* test). Enrichment analysis of MucR1-target genes for each replicons (**C**) and core/accessory subsets (**D**). Subset I (genus core), subset II (*S. fredii* core excluding subset I), subset III (genes shared by three closely related strains SF45436, SF25509 and HH103 but excluding subset I and II), subset IV (SF45436-specific genes). Significant enrichment/depletion is indicated (*, *P* value < 0.05; **, *P* value < 0.01; ***, *P* value < 0.001; Fisher’s exact test).

It is noteworthy that MucR1 targets are more intensively distributed among accessory genes of *S. fredii* (the third ring in Fig. 2C, gene content excluding species core genes) and plasmids or genomic regions with GC% lower than the average (the fourth ring in Fig. 2C). These global pictures are statistically verified in Fig. 4. AT% of target genes is generally higher than that of non-target genes (*t*-test, *P* values < 0.001) within individual replicons (chromosome, chromid pSFb, symbiosis plasmid pSFa, and two accessory plasmids pSFd/pSFe; Fig. 4A) or core/accessory subsets of different conservation levels ranging from genus core (Subset I) to SF45436-specific genes (Subset IV) (Fig. 4B). As high AT% is a characterized feature of foreign genes in Proteobacteria [70], these results imply MucR1 as a global regulator associated with foreign genes on different replicons and across different conservation levels.

This view is in line with a significant enrichment of MucR1 target genes on chromid (pSFb; Fisher’s exact test, *P* value < 0.05), symbiosis plasmid (pSFa; Fisher’s exact test, *P* value < 0.001) and an accessory plasmid pSFe (Fisher’s exact test, *P* value < 0.01) whereas depletion on chromosome (Fisher’s exact test, *P* value < 0.001) (Fig. 4C). On the other hand, targets are significantly enriched in the less conserved strain-specific Subset IV (Fisher’s exact test, *P* value < 0.001) whereas depleted in the more conserved genus core Subset I (Fisher’s exact test, *P* value <  0.001). Moreover, peaks with higher MucR1 recruitment levels were more frequently found on plasmids (Supplementary Fig. S3A) and associated with genes belonging to the less conserved subset IV (Supplementary Fig. S3B). In short, these findings suggest a preference of MucR1 to less conserved strain-specific foreign genes of higher AT content.

### MucR1 down regulates its AT-rich targets with predisposed high transcription potential

ATP and UTP are characterized by lower energy cost and more abundant over GTP and CTP [71], and there is experimental evidence suggesting that cell-level selection drives the genomes of extrachromosomal replicons toward higher A + T contents [72]. There is a pervasive mutational bias from GC toward AT in diverse bacteria [73]. However, an earlier test of potential fitness effects of AT content showed that, independent of adaptive codon use, artificial induction of AT-rich mRNA synonymous variants of two non-native genes (GFP and a phage DNA polymerase) in *E. coli* led to lower growth rates compared to induction of GC-rich variants [74]. Therefore, AT-rich genes seem to be better candidates to be banked in bacterial pangenome, but should be tightly regulated.

**Fig. 5:**
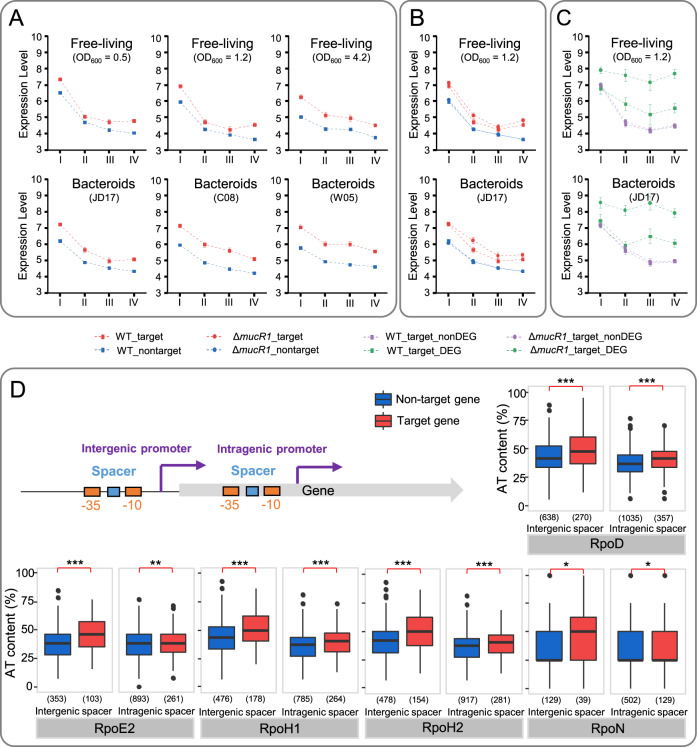
MucR1 down regulates its highly transcribed target genes across conservation levels. **A** MucR1 target genes were generally transcribed at a higher level than non-target genes under both free-living (TY medium; OD_600_ = 0.5, 1.2 and 4.2) and symbiotic conditions (Bacteroids from nodules of cultivated soybeans JD17 and C08, and wild soybean W05). **B** Average transcription level of MucR1 target genes was higher in the *mucR1* mutant than in SF45436. **C** A subset of MucR1 target genes were differentially expressed in a condition-dependent manner. **A**–**C** I–IV along the horizontal axis represent pangenome subsets of different conservation levels as defined in Figs. 2–4. **D** AT% of spacer sequences between −10 and −35 elements of predicted intergenic and intragenic promoters of different sigma factors. The numbers of spacers identified and analyzed for target and non-target genes are shown in brackets. *, *P* values < 0.05; **, *P* values < 0.01; ***, *P* values < 0.001 (Student’s *t* test).

To further investigate transcriptional characteristics of MucR1 target genes, we analyzed RNA-seq data from SF45436 or its *mucR1* mutant samples collected under various free-living and symbiotic conditions [21, 31]. Average transcriptional levels of both target and non-target genes decrease with the conservation levels from genus core to strain-specific genes (Fig. 5A), while AT content increases from genus core to strain-specific genes (Fig. 4B). Therefore, newer pangenome members of higher AT content are more likely down-regulated before successful integration with the existing regulatory network. This is supported by a genome scale mapping of chromosomal positions of high and low transcription propensity in *E. coli* using barcoded reporters [75], in which it was revealed that transcriptional level has negative correlation with AT content fraction.

The average transcription level of MucR1 target genes is generally higher than that of non-target genes under both free-living (TY medium; OD_600_ = 0.5, 1.2, and 4.2) and symbiotic (bacteroids from nodules of cultivated soybeans JD17 and C08, and wild soybean W05) conditions within each conservation levels (Fig. 5A) and replicons (Supplementary Fig. S4A). In the *mucR1* mutant, average transcription level of MucR1 target genes is higher than in the wild-type SF45436 (Fig. 5B and Supplementary Fig. S4B), particularly for those target genes (free-living, 144; bacteroids, 170) significantly differentially expressed (DEGs) between two strains (Fig. 5C and Supplementary Fig. S4C). It is intuitively striking that MucR1 target genes are generally transcribed higher than the non-target genes in the wild-type strain (Fig. 5A). Recently, it has been demonstrated that foreign genes are characterized with a higher AT content in their spacers between −10 and −35 elements than canonical promoters, which enhances the interaction of RNA polymerase with this DNA region and leads to a higher gene transcription level than those controlled by canonical promoters [76]. In line with this view, AT content of spacer sequences between −10 and −35 elements associated with MucR1 target genes is significantly higher than non-target ones regardless of whether predicted spacers are intergenic or intragenic (Fig. 5D). This phenomenon is sigma factor (RpoD, RpoE2, RpoH1, RpoH2 and RpoN) independent (Fig. 5D). Therefore, MucR1 has been recruited to down regulate AT-rich foreign genes which are predisposed to be highly transcribed (Fig. 5B–D), though this does not mean a complete repression (Fig. 5A).

### MucR1 recruitment levels increase with the number of periodic repeats of Ts and AT content

**Fig. 6:**
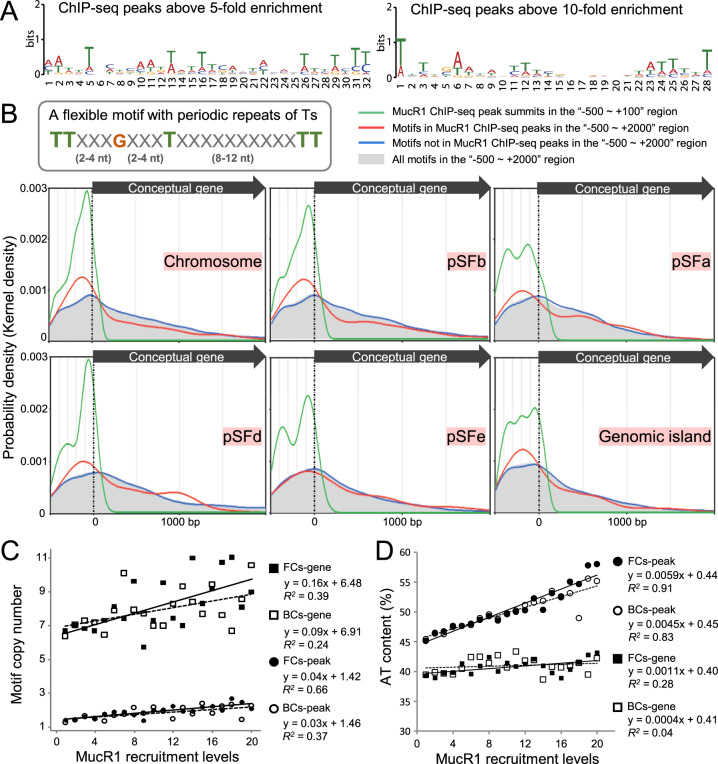
MucR1 targets are enriched in AT-rich sequences. **A** In silico deduced MucR1-binding motif within the 200 bp central region of ChIP-seq peaks with different enrichment folds compared to the input DNA. **B** Biased distribution of a flexible motif with periodic repeats of Ts in promoter regions across replicons and genomic islands (the black arrow indicates that only motifs within coding regions were considered in the “+1 ~ +2000” region to avoid interference by downstream intergenic sequences of those genes shorter than 2000 bp). Distribution of ChIP-seq peak summits within the “−500 ~ +100” region is shown for comparison. **C** Relationship between the number of the flexible motif (**B**) in individual ChIP-seq peaks (within the 200 bp central region; filled and open circles) or peak-associated genes (filled and open boxes) and MucR1 recruitment levels in the corresponding ChIP-seq peaks (horizontal axis). **D** Relationship between AT% of individual peaks (within the 200 bp central region; filled and open boxes) or peak-associated genes (filled and open boxes) and MucR1 recruitment levels in the corresponding ChIP-seq peaks (horizontal axis). FCs free-living ChIP-seq, BCs bacteroid ChIP-seq.

Structure of bacterial DNA impacts multiple cellular processes including binding mechanisms of various transcriptional factors such as the intensively studied xenogeneic silencers [56, 77]. AT-rich DNA is characterized by its more electronegative minor groove than GC-rich DNA, resulting higher affinity of AT-rich DNA for xenogeneic silencers H-NS/Lsr2/MvaT with positively charged residues (usually lysine and arginine) [36]. As to MucR/Ros, four basic regions individually harboring two to three arginine/lysine residues are essential for DNA binding [78], and MucR2 carrying a frameshift mutation in one of the basic region is not functional in *S. fredii* [31]. An in silico motif scanning analysis revealed that the putative MucR1 motifs are fairly degenerate and vary when subsets of peaks with different MucR1 recruitment levels (enrichment folds) were used (Fig. 6A). These flexible patterns have a property of 10–11 bp periodic repeats of T or TT (Fig. 6A), which have been defined as the class A flexible patterns and hypothesized as a putative new category of protein-DNA interaction sites [48]. The structure of the DNA-binding domain of Ros (Ros87) has been obtained [33], to which MucR1 shows 83% identity in the same domain and shares all conserved key residues involved in DNA binding [9]. Recently, a Ros87-DNA docking model and EMSA demonstrated that two Ts with 11 bp interval and an internal G are required for high affinity interaction between Ros87 and DNA [49]. Interestingly, the high affinity sequence targeted by Ros87 [49] shares the same signature as the typical class A flexible patterns [48] which are shown in Fig. 6B (TTXXXGXXXTXXXXXXXXXXTT). Genome wide analysis revealed that a higher probability density of this flexible motif (Fig. 6B) can be found in MucR1 ChIP-seq peaks than the other regions across different replicons and genomic islands (Fig. 6B). A significant positive correlation between the MucR1 recruitment level and the copy number of this flexible motif in ChIP-seq peaks (the 200 bp central region) was observed (Fig. 6C; Pearson’ rho is 0.81 and 0.61 for free-living and bacteroid ChIP-seq data, respectively; *P* values < 0.01). The importance of *cis* element variation is supported by a recent study on H-NS, in which with or without binding signals of xenogeneic silencer H-NS can partially explain a wild range of expression variability of a foreign gene that was randomly inserted into genomes of a population of *E. coli* [79]. In line with this scenario, a great variation in MucR1 recruitment levels was observed within individual replicons or pangenome subsets of *S. fredii* (Supplementary Fig. S3). The proportion of genus core among target genes negatively correlated with MucR1 recruitment levels of associated peaks (Supplementary Fig. S3B), implying that the more conserved a gene is the less likely being strongly targeted by MucR1.

Similar to other xenogeneic silencers, a positive correlation between AT% and the protein recruitment level was also found for MucR1 (Fig. 6D; Pearson’ rho is 0.96 and 0.93 for free-living and bacteroid ChIP-seq data, respectively; *P* values < 0.001). These findings imply an intriguing model in which the recruitment levels of MucR1 may gradually decay with the decrease of AT content and the erosion of the typical flexible patterns harboring periodic repeated Ts. This model fits well with the view that AT-rich signature of foreign DNA fragments will be progressively erased in the longterm evolution of bacterial genomes [70], and may allow subsequent selection of cells with the optimal expression levels of foreign genes, given adaptive benefit was explored in particular niches.

**Fig. 7:**
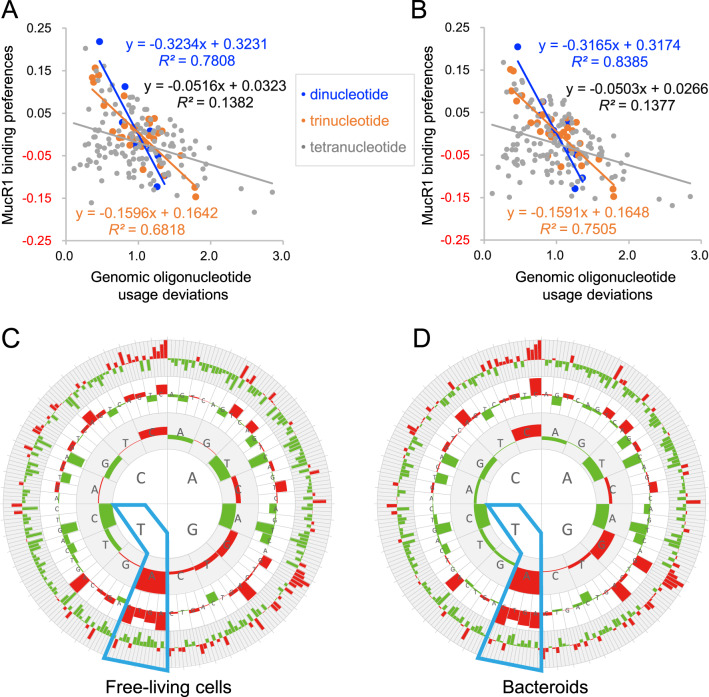
MucR1 ChIP-seq peaks are enriched with TpA steps. **A**, **B** Relation between whole genome usage deviations and MucR1 binding preferences of oligonucleotide under free-living/bacteroid conditions. The height of red/green bars represents the positive/negative coefficient (Spearman’s rho) between the oligonucleotide usage deviations and MucR1 recruitment levels in ChIP-seq peaks identified in free-living cells (**C**) or bacteroids (**D**). Bar scales for free-living cells/bacteroids: −0.129~0.205/−0.123~0.218 (dinucleotide), −0.148~0.151/−0.147~0.156 (trinucleotide), −0.168~0.168/−0.182~0.203 (tetranucleotide). Oligonucleotide compositions of the 200 bp central regions of ChIP-seq peaks were analyzed.

The highest-affinity sequences for known convergently evolved xenogeneic silencers H-NS, MvaT, Lsr2 and Rok harbor TpA dinucleotides (often referred as “steps”) [16, 80]. This phenomenon can be partially explained by the fact that TpA steps, among all dinucleotide steps, confer the most flexibility on DNA, facilitating accommodation of these xenogeneic silencers in the minor groove [36]. To determine the potential relationship between oligonucleotide composition of ChIP-seq peaks (tetranucleotide, trinucleotide and dinucleotide) and MucR1 recruitment levels (enrichment fold), Spearman’s rho coefficient was calculated. The strongest correlation (rho = 0.389 and 0.424, *P* value = 1.0E−54 and 8.3E−48 for FC and BC peaks, respectively) was found between MucR1 recruitment levels and oligonucleotides composed of A and T. Considering that the inherent bias in genome sequence might have impact on this analysis, oligonucleotide deviation values [52] instead of content values were further analyzed (Fig. 7A, B). A strong linear relationship between the recruitment level and dinucleotide (*R*^2^ = 0.78 for FC; *R*^2^ = 0.84 for BC) or trinucleotide (*R*^2^ = 0.68 for FC; *R*^2^ = 0.75 for BC) was observed for both FC (Fig. 7A) and BC (Fig. 7B) samples. Moreover dinucleotide TpA (TA step) and trinucleotides containing TpA had the highest positive correlation with the recruitment level (Fig. 7C, D), though the correlation seems to be weak (rho = 0.205 and 0.218, *P* value = 1.8E−15 and 5.9E−13 for FC and BC peaks, respectively).

## Conclusion

**Fig. 8:**
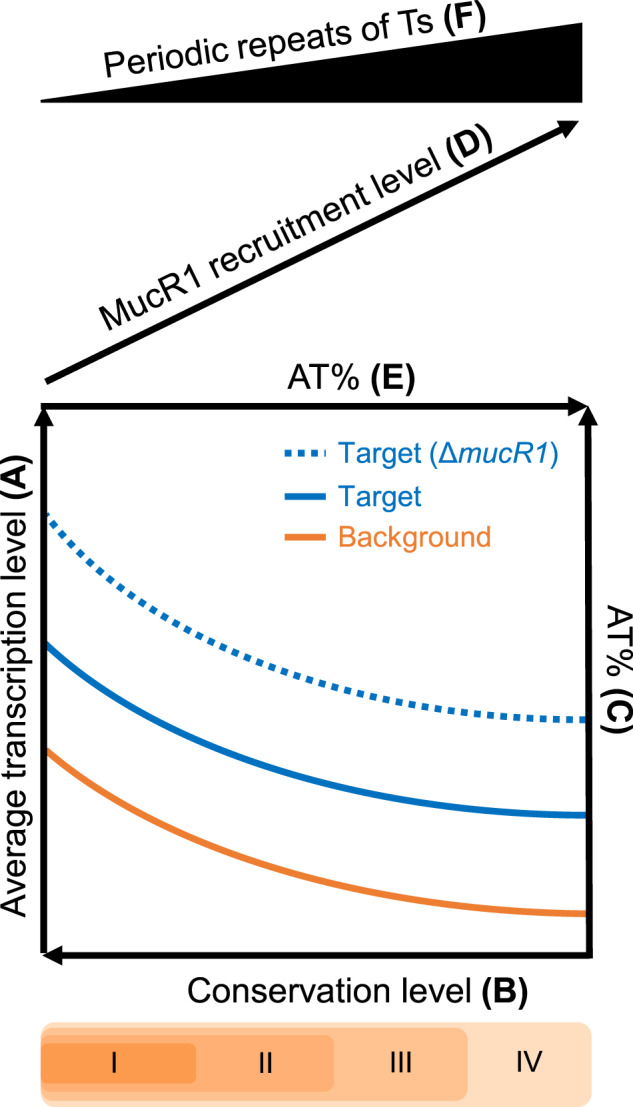
Schematic model showing the maintenance and recruitment of foreign AT-rich genes mediated by MucR1 during adaptive evolution. The average transcription level (**A**) is positively correlated with the conservation level of genes (**B**), subsets of which can be targeted by MucR1 and have higher AT% (**C**). These AT-rich MucR1 targets are predisposed to be transcribed at levels higher than genomic background even with a functional MucR1 (solid blue line). In the Δ*mucR1* mutant, MucR1 targets were generally up-regulated (dashed blue line), though condition-dependent transcriptional profiles can be observed for genus or species core genes, implying an integration with the existing regulation network. The recruitment level of MucR1 (**D**) decreases with higher GC% of targets (**E**) and their lower number of a flexible motif characterized by periodic repeats of Ts (**F**), fitting well with the view that AT-rich signature of foreign DNA can be progressively erased during adaptive evolution.

Horizontal gene transfer (HGT) plays a critical role in increasing bacterial genetic diversity and the ability of exploring previously inaccessible niches [5, 70, 81] while challenging the regulation integrity. Despite several convergently evolved xenogeneic silencers including H-NS, MvaT, Lsr2 and Rok have been discovered, it remains largely unexplored about the recruitment mechanisms of beneficial foreign genes. By focusing on the facultative microsymbiont *S. fredii*, this work reveals that the more conserved a gene is, the lower its AT content (Fig. 4B) and the higher its average transcription level (Fig. 5A). MucR1, a conserved zinc-finger regulator in α-proteobacteria, can extensively binds AT-rich regions across pangenome subsets of different conservation levels and replicons (Figs. 2C and 4A, B). MucR1 targets with known functions in environmental adaptation and symbiosis are enriched in genus and species core genes implying successful integration of these AT-rich genes (Fig. 3), while ChIP-seq peaks of higher MucR1 recruitment levels are associated with less conserved strain-specific genes of higher AT content and lower transcription levels (Figs. 4D and 5A, Supplementary Fig. S3B). Within each conservation levels, MucR1 down regulates its AT-rich targets which are predisposed to be highly transcribed (Fig. 5A) due to their higher AT% than non-target genes in the spacer sequences between −35 and −10 elements of promoters which enhance transcription efficiency [76] (Fig. 5C, D). A key mechanism facilitating the recruitment of potential beneficial AT-rich foreign genes may at least partially involve the erosion of MucR1-binding sites characterized by periodic repeats of Ts (Fig. 6C). In line with the view that AT-rich signature of foreign DNA can be progressively erased during adaptive evolution [8, 70], this work suggests that MucR directly represses AT-rich foreign genes with predisposed high transcription potential while the progressive erosion of its target sites facilitates the integration of beneficial foreign genes into the regulation network. This adaptive regulation mechanism summarized in Fig. 8 can be common for prokaryotes carrying a xenogeneic silencer managing the adaptive pangenome when exploring various niches.

## Supplementary information

Supplementary Figures S1-S4

Table S1

Table S2

Table S3

## Data Availability

Raw sequence data from our RNA-seq and ChIP-seq analyses can be accessed via NCBI Sequence Read Archive (﻿PRJNA389250, ﻿PRJNA302586 and PRJNA302588).
