# Supplementary material for: The zinc-finger bearing xenogeneic silencer MucR in α-proteobacteria balances adaptation and regulatory integrity
Source: ISME J. 2021 Sep 28;16(3):738–49. doi: 10.1038/s41396-021-01118-2 (PMC8857273; doi:10.1038/s41396-021-01118-2)
Supplement: Supplementary file 1 — Supplementary Figures S1-S4 [file 41396_2021_1118_MOESM1_ESM.pdf]

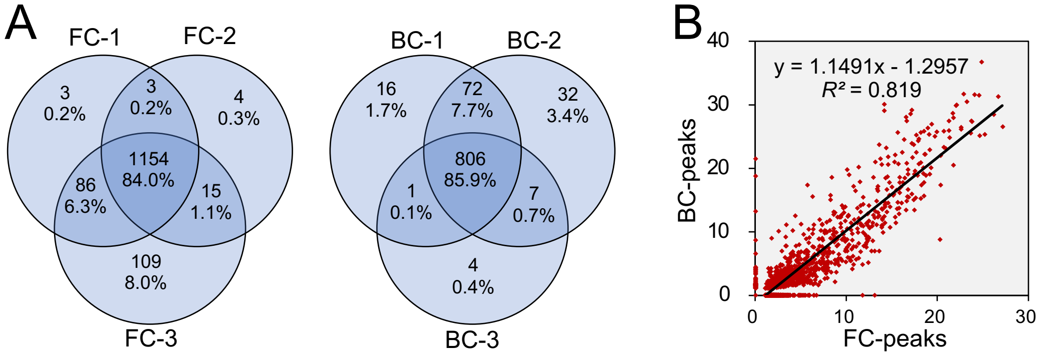

Figure S1. Venn diagram (A) and regression analysis (B) showing MucR1 ChIP-seq peaks identified in this study. FC, free-living cells; BC, bacteroids. Three biological replicates were performed.

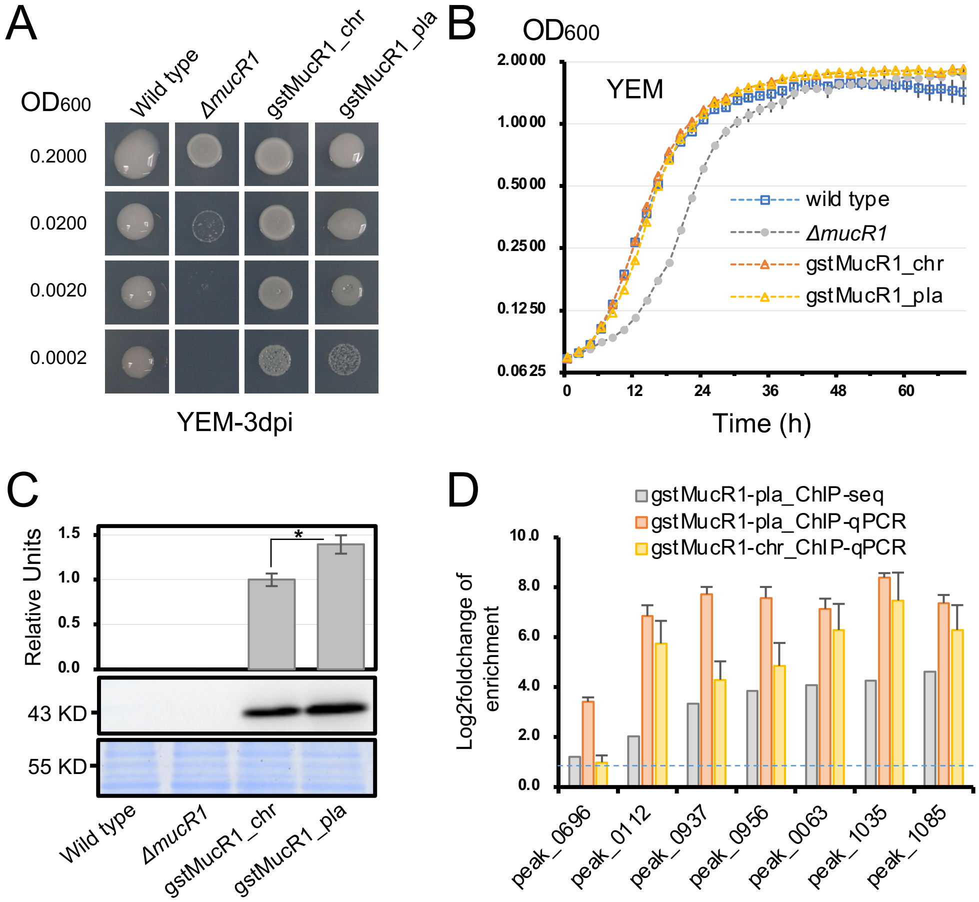

Figure S2. Comparative analysis of gstMucR1_pla and gstMucR1_chr. (A) EPS production of *S. fredii* strains on YEM agar plate. The gstMucR1_pla (Δ*mucR1* complemented by the pBBR1MCS-2::*PmucR1-gst-mucR1*) and the gstMucR1_chr (Δ*mucR1* complemented by the *gst-mucR1 in situ*). Each colony was dotted with 2 μL rhizobial suspension with gradient optical density at OD_{600} as indicated. **(B)** Growth curves of *S. fredii* strains cultured in YEM broth medium monitored by Bioscreen C Microbiology Reader. **(C)** Quantitative analysis of expression level of GST-MucR1 fusion protein by Western blot using anti-GST. The protein level of GST-MucR1 in gstMucR1_pla is approximately 1.4-fold of that in gstMucR1_chr (*, P value < 0.01, student's *t*-test). **(D)** ChIP-qPCR analysis of GST-MucR1 enrichment at candidate peaks with different foldchanges identified by ChIP-seq in different strains under free-living condition (TY culture reach to OD_{600} = 1.2). DNA fragments associated with all these peaks are significantly enriched in ChIP samples prepared from both gstMucR1_chr and gstMucR1_pla strains (foldchange > 1, P values < 0.01, $^{\Delta\Delta}$Ct method, student's *t*-test). Consistent with many other early studies, MucR1 can negatively regulate its expression by binding to its own promoter region (peak_0112).

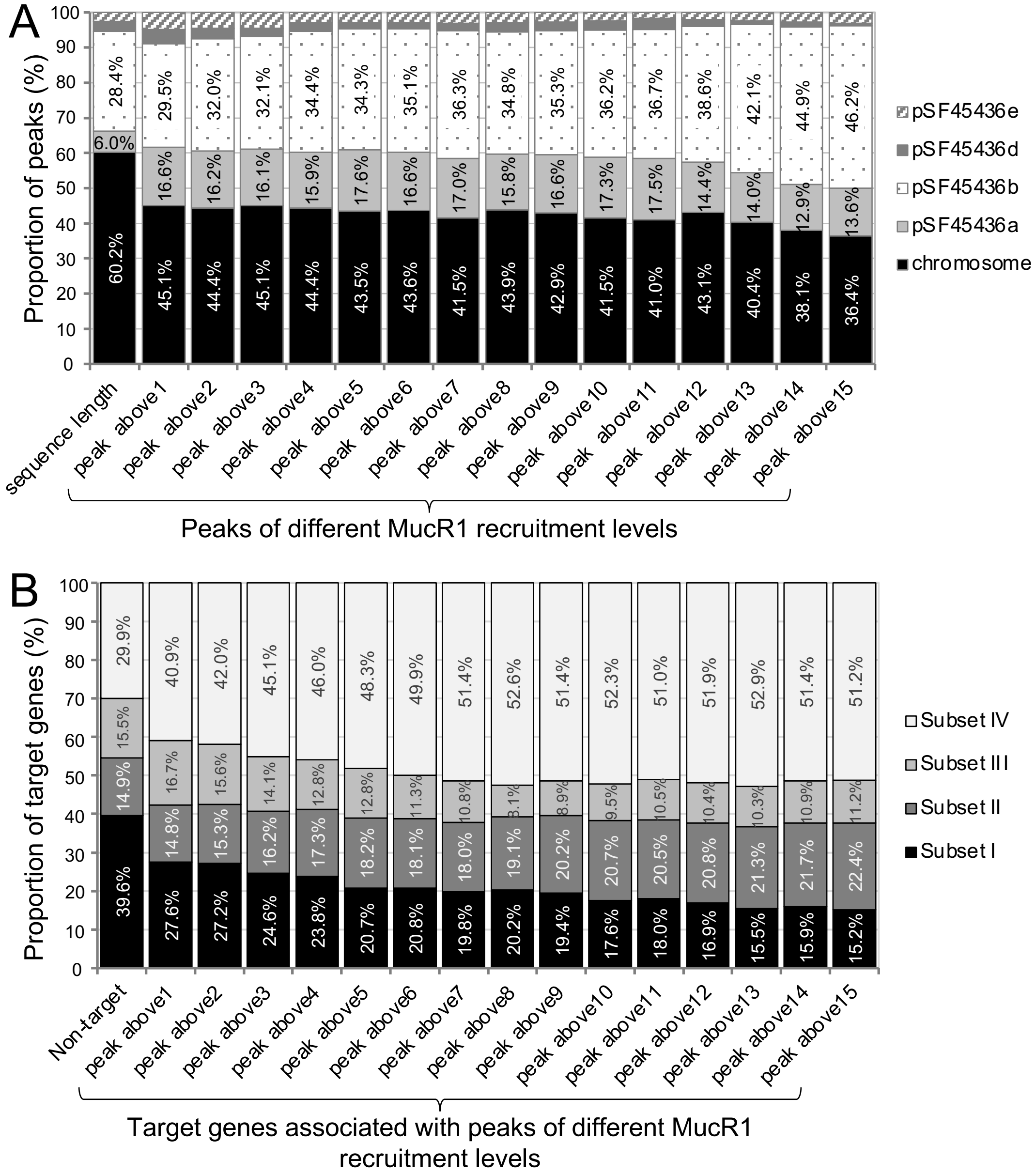

Figure S3. Peaks with higher MucR1 recruitment levels were more frequently found on plasmids (A) and associated with less conserved subset IV (B). Peaks enriched by the indicated folds were studied. Sequence length in (A) indicates the relative length of five replicons. Proportion of non-target genes is shown in (B) for comparison.

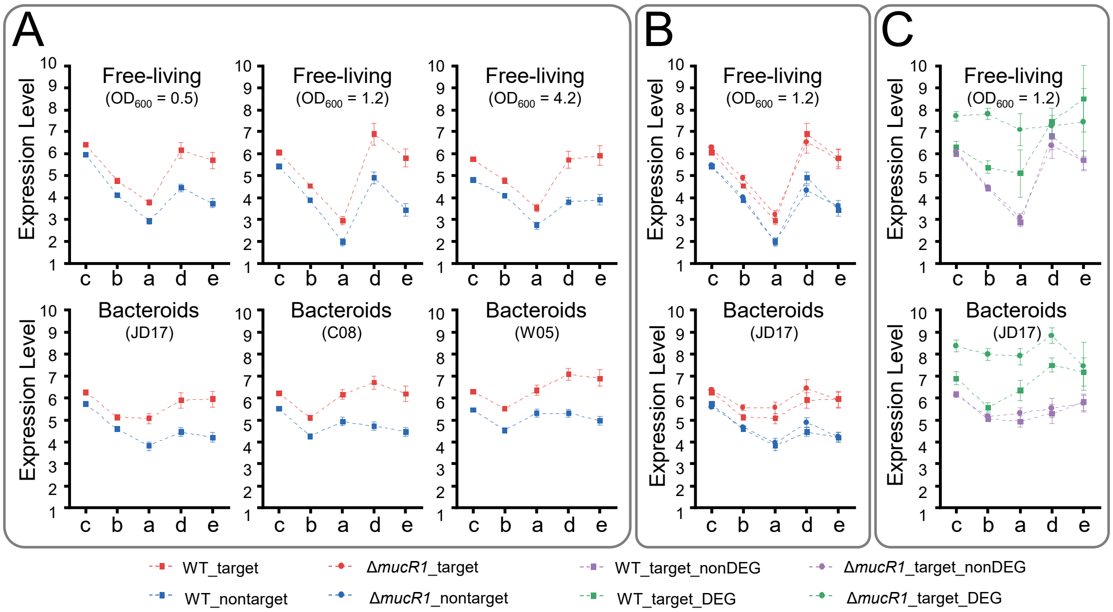

Figure S4. MucR1 down regulates its highly transcribed target genes on different replicons. (**A**) MucR1 target genes were generally transcribed at a higher level than non-target genes under both free-living (TY medium; OD_{600} = 0.5, 1.2 and 4.2) and symbiotic conditions (Bacteroids from nodules of cultivated soybeans JD17 and C08, and wild soybean W05). (**B**) Average transcription level of MucR1 target genes was higher in the *mucR1* mutant than in SF45436. (**C**) A subset of MucR1 target genes were differentially expressed in a condition-dependent manner.
