# Supplementary material for: The zinc-finger bearing xenogeneic silencer MucR in α-proteobacteria balances adaptation and regulatory integrity
Source: ISME J. 2021 Sep 28;16(3):738–49. doi: 10.1038/s41396-021-01118-2 (PMC8857273; doi:10.1038/s41396-021-01118-2)
Supplement: Supplementary file 4 — Table S3 [file 41396_2021_1118_MOESM4_ESM.pdf]

Table S3 Responses of wild soybean plants to inoculation of *S. fredii* CCBAU45436 and its derivatives

Strain	Shoot height (cm/plant)	Chlorophyll content (SPAD)	Nodule number (per plant)	Nodule fresh weight	
				(mg/plant)	(mg/nodule)
Uninoculated control	12.7 ± 1.3*	17.8 ± 0.6*	-	-	-
Wild type	26.3 ± 2.1	45.0 ± 0.9	15.4 ± 2.6	89.7 ± 8.4	6.7 ± 1.0
Δ*mucR1*	15.9 ± 1.4*	19.7 ± 0.9*	23.7 ± 1.2*	116.8 ± 7.7*	5.0 ± 0.3
Δ*mucR1*+GST-MucR1_chr	29.7 ± 4.7	43.3 ± 0.9	15.6 ± 2.0	75.9 ± 9.8	4.9 ± 0.5
Δ*mucR1*+GST-MucR1_pla	25.1 ± 2.0	43.5 ± 0.8	14.8 ± 1.1	75.8 ± 4.6	5.3 ± 0.4

Note: Average ± standard error of the means scored from 7~11 plants. *, P value < 0.001, significant difference compared with the wild-type strain (student's *t*-test).
